# Light-Induced Stomatal Opening Is Affected by the Guard Cell Protein Kinase APK1b

**DOI:** 10.1371/journal.pone.0097161

**Published:** 2014-05-14

**Authors:** Nagat S. Elhaddad, Lee Hunt, Jennifer Sloan, Julie E. Gray

**Affiliations:** Department of Molecular Biology and Biotechnology, University of Sheffield, Sheffield, United Kingdom; University College Dublin, Ireland

## Abstract

Guard cells allow land plants to survive under restricted or fluctuating water availability. They control the exchange of gases between the external environment and the interior of the plant by regulating the aperture of stomatal pores in response to environmental stimuli such as light intensity, and are important regulators of plant productivity. Their turgor driven movements are under the control of a signalling network that is not yet fully characterised. A reporter gene fusion confirmed that the Arabidopsis *APK1b* protein kinase gene is predominantly expressed in guard cells. Infrared gas analysis and stomatal aperture measurements indicated that plants lacking APK1b are impaired in their ability to open their stomata on exposure to light, but retain the ability to adjust their stomatal apertures in response to darkness, abscisic acid or lack of carbon dioxide. Stomatal opening was not specifically impaired in response to either red or blue light as both of these stimuli caused some increase in stomatal conductance. Consistent with the reduction in maximum stomatal conductance, the relative water content of plants lacking APK1b was significantly increased under both well-watered and drought conditions. We conclude that APK1b is required for full stomatal opening in the light but is not required for stomatal closure.

## Introduction

The activity of several protein kinases has been shown to be important in mediating stomatal responses to environmental stimuli, including light intensity, pathogens, atmospheric carbon dioxide concentration and the drought hormone abscisic acid (ABA). Well-characterised Arabidopsis guard cell kinases include HIGH LEAF TEMPERATURE (HT1), OPEN STOMATA 1 (OST1), phototropins PHOT1 PHOT2, PKS5, BLUE LIGHT SIGNALLING 1 (BLUS1) and calcium-dependent protein kinases CPK 3, 6 21 and 23, which are required for stomatal closure responses to atmospheric CO_2_ concentration [1], [2] and abscisic acid (ABA) [3], [4], [5] and for the stomatal opening response to blue light [6], [7], [8]. Additionally a lectin type receptor kinase (LecRK-V.5) is important for control of stomatal aperture in response to pathogens [9].

The Arabidopsis genes *Arabidopsis protein kinase 1a (APK1a; AT1g07570*) and *APK1b* (*At2g28930*) encode protein kinases with peptide sequences that are 90% identical. The encoded proteins phosphorylate tyrosine, serine and threonine residues *in vitro* [10] but the nature of their *in vivo* substrate(s) have not been investigated. Beyond their kinase activity their function is unknown, although transcriptomics experiments suggest that *APK1a* and *APK1b* are predominantly expressed in guard cells [11] (**Figure S1**). Homology searching suggests that these genes are members of the receptor-like cytoplasmic kinase VII subfamily [12]. The role of this subfamily of kinases has not previously been investigated in stomatal responses. The best characterised member of the VII subfamily is the Brassica, *M* locus protein kinase (MLPK) which acts in cells of the stigma, downstream of the *S* receptor kinase to mediate rejection of self-incompatible pollen [13]. MLPK has sequence similarities with receptor-like kinases but like APK1a and APK1b, it has no apparent signal sequence or transmembrane domain and is believed to attach to the plasma membrane via *N*-myristoylation [12]. There are 46 members of the kinase VII subfamily encoded in the Arabidopsis genome. APK1a and APK1b have closest homology to *Botrytis Induced Kinase 1* (*BIK1*), lack of which results in severe susceptibility to necrotrophic fungal pathogens but does not impair responses to the bacterial pathogen *Pseudomonas syringae* [14].

In this study we demonstrate that *APK1b* is expressed predominantly in guard cells. We report experiments with two Arabidopsis T-DNA insertion lines lacking the *APK1b* gene transcript, which indicate that this protein kinase is required for full light-induced stomatal opening.

## Results

### APK1b Promoter Directs Expression to Guard Cells

**Figure 1:**
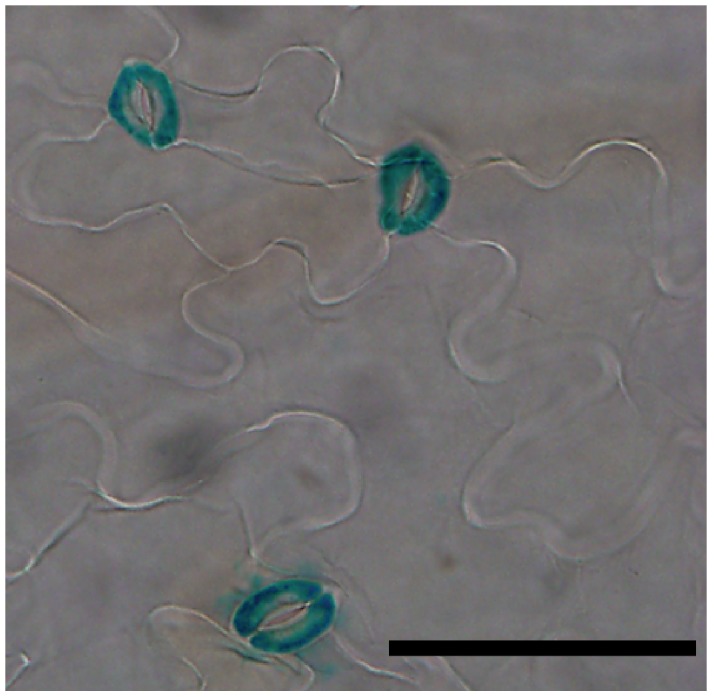
APK1b promoter direct GUS expression to the guard cells. GUS histochemical localisation of expression directed by the *APK1b* upstream gene promoter in leaves of 3 week old seedlings. Scale bar = 50 µm.

To determine the cellular expression pattern, *P_APK1b_: GUS-GFP* seedlings were histochemically stained. *GUS* expression was strongly localized to guard cells (**Figure 1**). GUS staining was particularly apparent in mature stomata of leaves with a lesser amount of GFP fluorescence detectable in some stomatal precursor cells (meristemoids or guard mother cells Figures S2A–C). Expression of P*_APK1b_: GUS-GFP* was also associated with the mature cauline leaves and sepals (**Figure S2**).

### The Effect of Changes in Light Intensity on *apk1b* Stomatal Conductance

**Figure 2:**
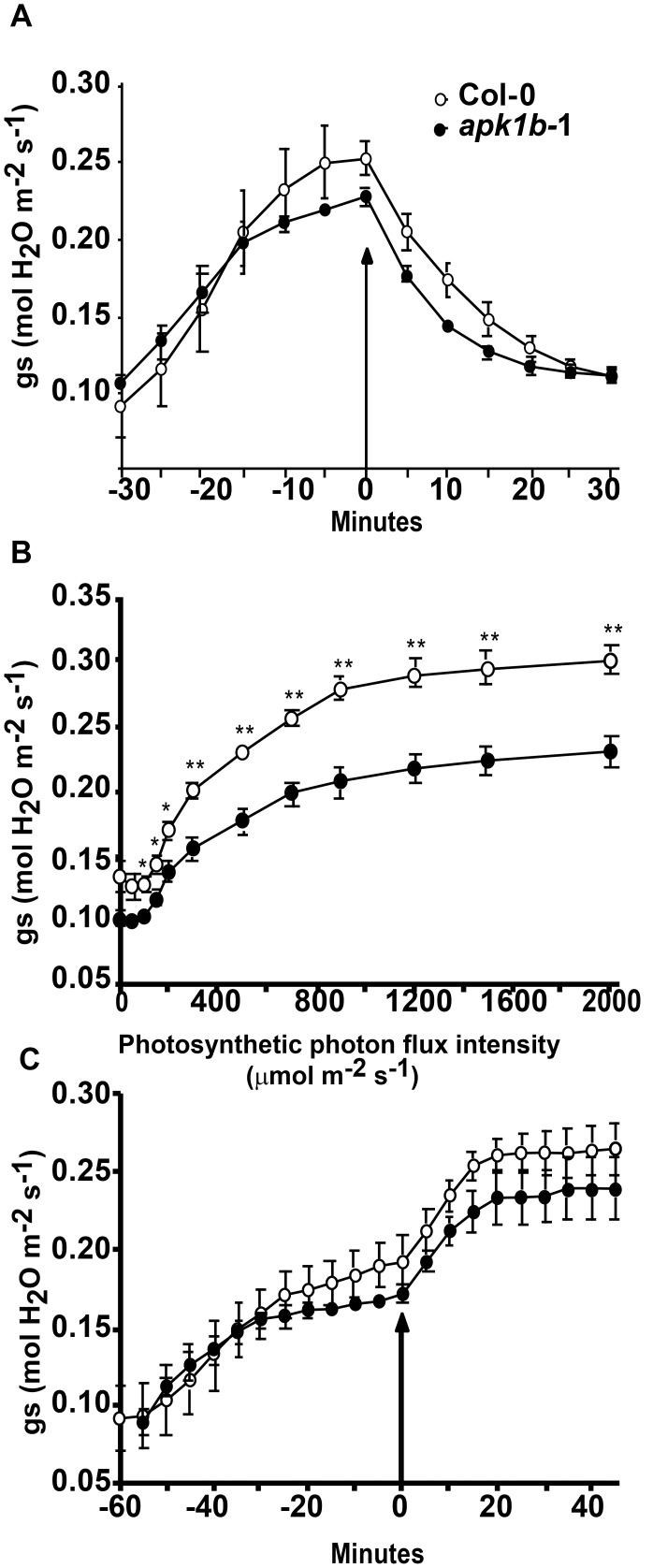
Stomatal conductances of *apk1b-1* are lower than control plants. Mean stomatal conductances of *apk1b-1* plant leaves compared to Col-0 control leaves. A: Leaves of plants exposed to saturating light for 30 min, then exposed to darkness, arrow indicates when light was switched off. B: Leaves exposed to increasing light intensities ranging from 0 to 2000 µmol photon m^−2 ^s^−1^. C: Leaves of plants exposed to red light for 60 min, then exposed to blue and red light, arrow indicates when blue light was switched on. Error bars represent the standard error. Values were statistically tested using unpaired t-tests and significant differences from Col-0 control are indicated (* = *p*<0.05, ** = p<0.01).

Two independent Arabidopsis T-DNA insertion lines, *apk1b-1* and *apk1b-2*, that disrupt the *APK1b* gene within the first exon and the first intron respectively, were identified and RT-PCR used to confirm that the wild-type gene transcript was not present in either plant genotype (**Figure S3**). Infrared gas analysis (IRGA) was used to investigate alterations in stomatal conductance in leaves of *apk1b-1* plants in response to light and dark stimuli. To investigate the effect of darkness on *apk1b-1*, IRGA readings were taken under saturating light (1500 µmol photon m^−2 ^s^−1^) for 30 minutes, then the light source was turned off and stomatal conductance measured every five minutes in darkness. Mutant plants had lower mean conductances than wild-type plants in this experiment following exposure to saturating light but none of the differences observed were significant (**Figure 2A**). Conductances appeared to reduce at a similar rate in the absence of light and both genotypes had reached minimum conductances after 30 minutes in darkness, suggesting that the lack of APK1b does not affect stomatal closure.

To investigate the effect of changing light intensity, plants were taken during the photoperiod and exposed to increasing light intensities in the IRGA chamber starting with 0 and finishing with 2000 µmol photon m^−2 ^s^−1^_._ Light intensity was increased by 50 µmol photon m^−2 ^s^−1^ every 5 minutes. At the start of the experiment, in darkness, the difference between *apk1b-1* mutant and control mean stomatal conductances was not significant. Both increased at higher light intensities but mean *apk1b-1* conductance remained lower than controls throughout the experiment. The differences in conductance observed were significant between 50 and 2000 µmol photon m^−2 ^s^−1^ (**Figure 2B**). Thus *apk1b-1* leaf stomatal conductance was lower than control plants over a range of light intensities but was not significantly different in dark-adapted plants, suggesting that the stomata of *apk1b-1* may be impaired in their ability to open in response to increasing light, but not in their ability to close in the dark. Both photosynthetic rate and intracellular CO_2_ were recorded in the IRGA chamber at the same time as stomatal conductance but neither were significantly different at any time point except for the response to 500 µmol photon m^−2 ^s^−1^ light (**Figure S4**) which was lower in *apk1b-1* than controls.

As stomata open in response to both blue and red light signals, we carried out IRGA to investigate whether either of these responses was specifically affected by the lack of APK1b. In the experiment shown in **Figure 2C** leaves were initially exposed to red light then after an hour, when stomatal conductances had stabilised, blue light was also applied. *apk1b-1* mean stomatal conductance at the start of the experiment (in the dark) was similar to controls but after thirty minutes exposure to red light, was consistently lower than controls, in line with the results presented above (in **Figure 2A and B**). However, as the difference in conductance between mutant and control plants was not significant at any time point, and *apk1b-1* stomatal conductances increased in response to both red light and blue light, the stomatal opening response to neither of these light wavelengths appeared to be specifically impaired.

### APK1b Mutants have Normal Stomatal Development

**Figure 3:**
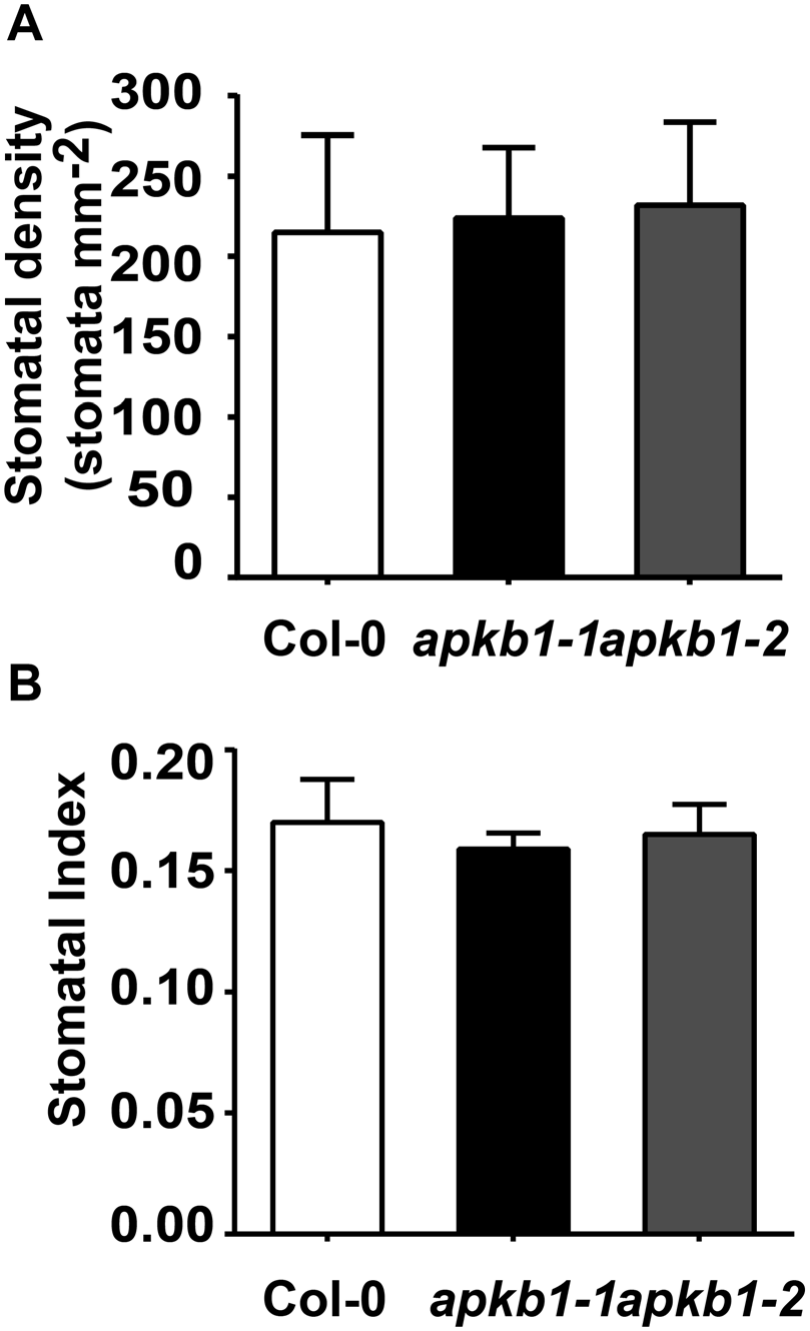
Stomatal density and index of *apk1b-1*and *apk1b-2* are similar to those of control plants. A: Stomatal density and B: stomatal index were calculated from the abaxial surface of fully expanded leaves. Error bars represent the standard error.

Epidermal cell counts were carried out to investigate whether *apk1b-1* leaves could display lower stomatal conductance as a result of reduced stomatal number. However, the stomatal index and density were similar for wild-type, *apk1b-1* and *apk1b-2* plants and there were no significant differences between genotypes (**Figure 3**). Nor were there any obvious differences in stomatal size or appearance. Mean stomatal aperture lengths of *apk1b-1, apk1b-2* and Col-0 were measured from epidermal peels and found to be 4.64, 4.84 and 4.77 µm respectively. These results suggest that the altered stomatal conductances described above may be due to defective stomatal aperture control in response to light, rather than defective stomatal development.

### The Effect of Light, ABA and CO_2_ on Stomatal Apertures

**Figure 4:**
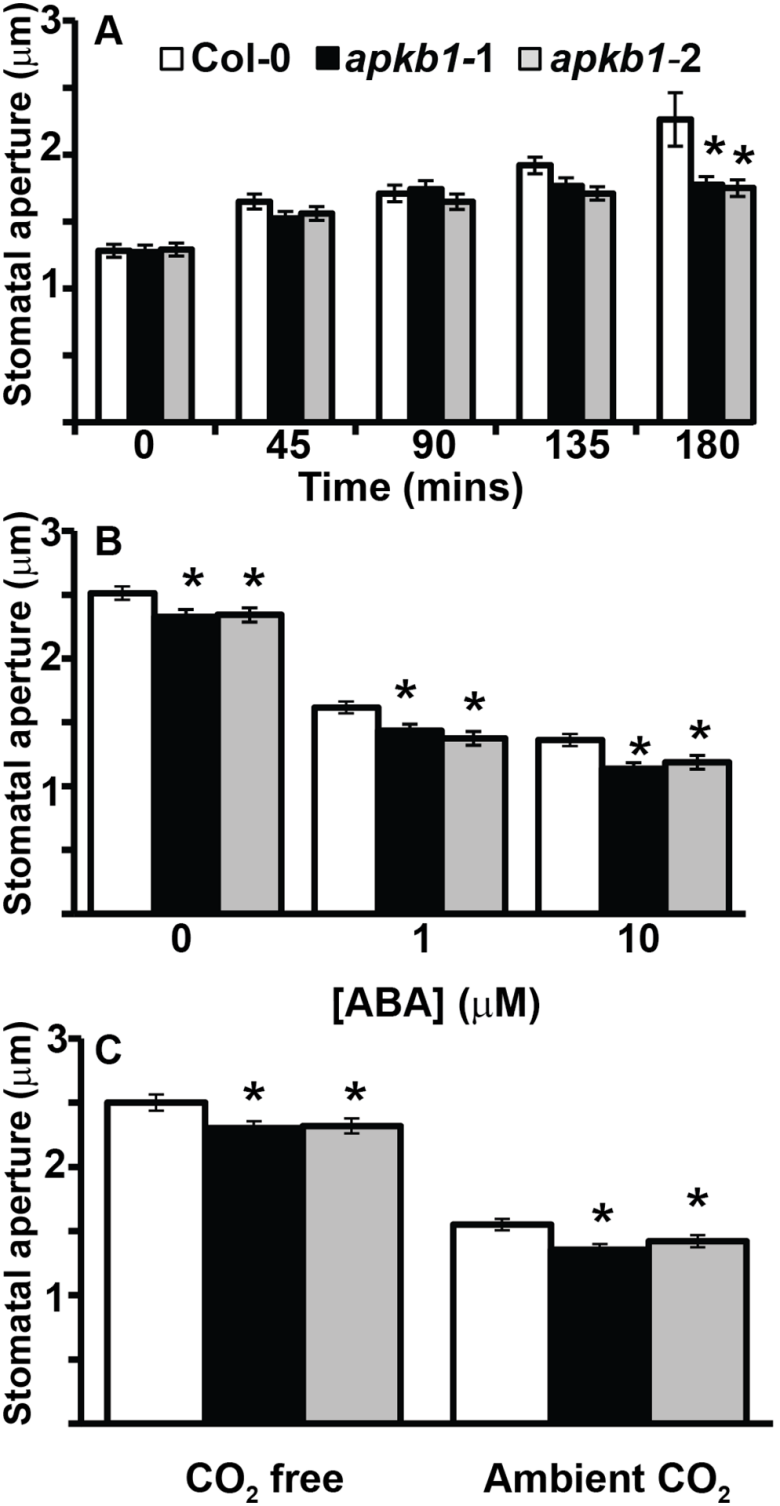
Stomata of plants lacking APK1b cannot open as wide in response to light but can close in response to ABA or CO_2_. The effect of A: light, B: ABA and C: CO_2_ on stomatal aperture measurements from *apk1b-1, apk1b-2* and control plants. Apertures were measured from detached leaf abaxial peels following treatment with stimuli as indicated. For the response to light shown in A. plants were dark-adapted and peels prepared in dim light at the start of the experiment. Significant differences from Col-0 control are indicated (* = p<0.05).

To determine whether reduced stomatal conductance could be due to an alteration in stomatal aperture control, direct measurement of *apk1b-1* and *apk1b-2* stomatal apertures was carried out following treatment of epidermal peels with differing environmental stimuli. Aperture changes in response to light, ABA and CO_2_ treatments were measured by microscopy. To investigate stomatal opening in response to light, plants were kept in the dark overnight; epidermal peels were taken from each genotype in dim-light and kept in resting buffer for an hour before being transferred to opening buffer in the light. The mean apertures of *apk1b* stomata were similar to controls in the dark-adapted samples at the start of the experiment. The apertures of wild-type and both *apk1b-1 and apkb-2* genotypes increased during exposure to light but *apk1b-1* and *apk1b-2* stomata did not open as wide as controls. The differences observed between control and *apk1b-1* and *apk1b-2* mean stomatal apertures were significant after 180 minutes exposure to light (p<0.05) (**Figure 4A**). This result is consistent with the *apk1b-1* reduced stomatal conductance in the light observed by IRGA (**Figure 2**) and further suggests that the stomata of *apk1b* mutant plants are defective in light-induced opening.

To investigate the inhibition of stomatal opening by ABA epidermal peels were incubated two hours in the light in opening buffer with differing concentrations of ABA and then stomatal apertures were measured. Both *apk1b* mutant lines had significantly lower mean stomatal apertures than controls when no ABA was added and following incubation with 1 or 10 µM ABA (**Figure 4B**). However, *apk1b-1* and *apk1b-2* stomata appeared to have similar sensitivity to ABA as controls at these ABA concentrations.

The response of stomatal apertures to CO_2_ concentration was studied by incubating epidermal peels for two hours in the light in opening buffer supplied (and pre-equilibrated) with either CO_2_ free air or ambient CO_2_ air before apertures were measured. Again *apk1b-1* and *apk1b-2* mean stomatal apertures were significantly smaller than control apertures under both conditions (**Figure 4C**). However, the magnitude of response to CO_2_-concentration was similar for all genotypes and the response of *apk1b* stomata to CO_2_ was not impaired. Together these results indicate that *apkb1* stomata are able to adjust their apertures to at least the same extent as control stomata in response to a variety of closure stimuli (darkness, exogenous ABA, and elevated CO_2_) but are unable to open as to the same extent in response to light.

### *apk1b* Plants have Reduced Transpiration and Increased Water Content

**Figure 5:**
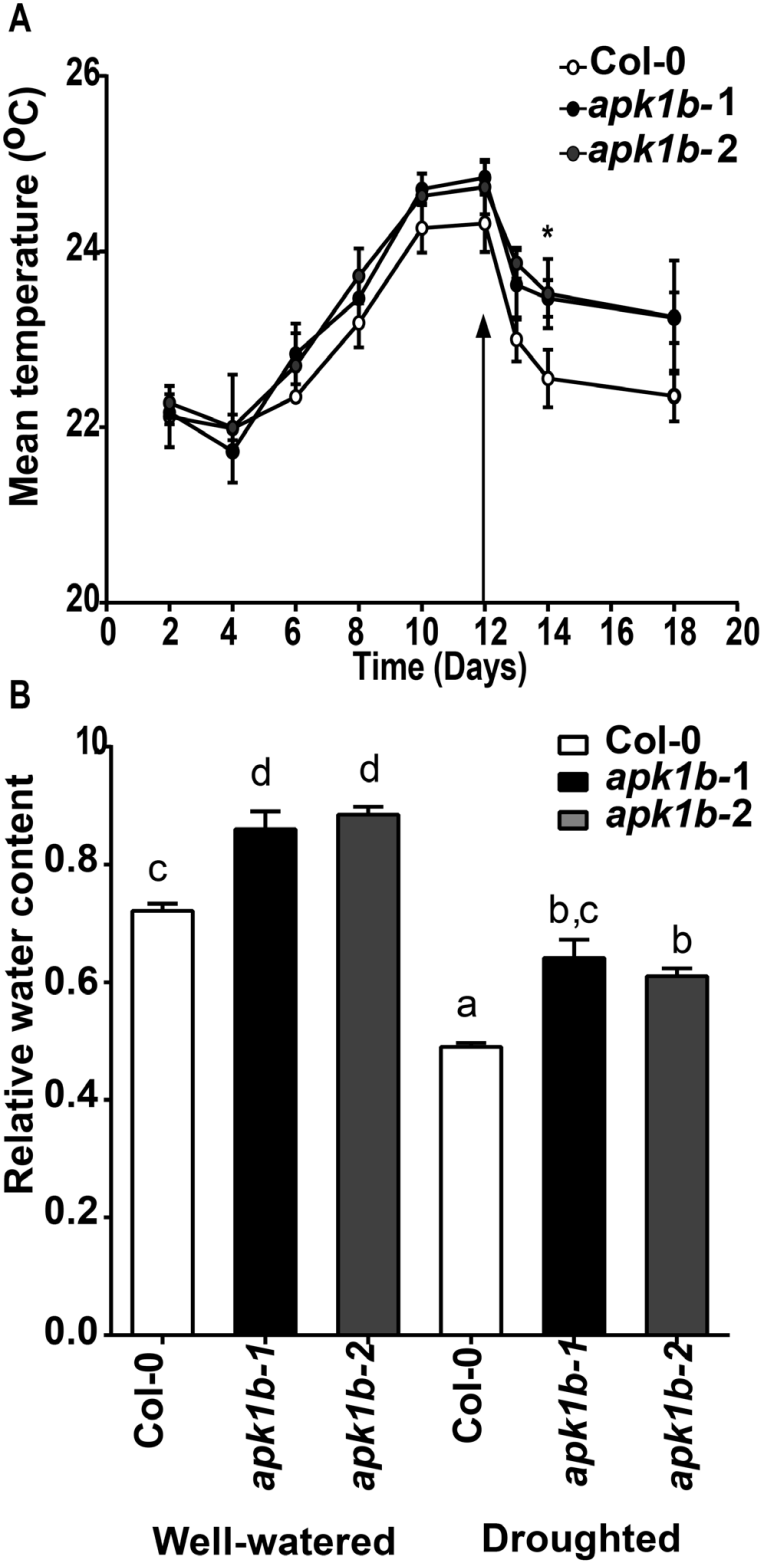
*apk1b-1* and *apk1b-2* have reduced levels of transpiration and increased relative water content in comparison to control plants. A: Temperature of the leaf surface following drought imposition for the time indicated was measured by infra-red thermography. The onset of watering is indicated by an arrow. Significant differences are indicated (* = p<0.05). B: Relative water content was calculated from measurements of well-watered plants and plants subjected to 7 days of drought. Letters indicate significance groups (p<0.05).

We investigated whether the reduced stomatal opening phenotype of the *apk1b* plants that we describe above, could have an effect on plant transpiration and plant water status by examining leaf temperature, and relative water content. Infra-red thermal imaging indicated that both *apkb1-1* and *apkb1-2* plants had higher mean day-time leaf temperatures (suggesting reduced evaporative cooling from transpiration) following severe drought treatment and subsequent re-watering. 2 days after re-watering following a period without water the mutant plant lines were both approximately 0.8°C hotter than control plants indicating a reduced level of transpiration (**Figure 5A**).

Both *apk1b-1 and apk1b-2* plants had significantly higher RWC than controls under either a normal watering regime or following seven days without watering, confirming that *apk1b-1* and *apk1b-2* are better able to conserve water than wild-type plants (**Figure 5B**). The increased leaf temperature and water content are both consistent with *apk1* stomata opening less in the light (**Figures 2** **and** **4**).

## Discussion

A number of transcriptomics experiments have suggested that the protein kinase encoded by *APK1b* is highly expressed in guard cells relative to other cell types [11], [15], [16]. In the current study it was demonstrated that the upstream promoter region of the *APK1b* gene directs expression predominantly in guard cells, and the potential role that *APK1b* may play in the control of stomatal apertures was explored. Gas exchange analysis revealed that plants lacking *APK1b* expression (*apk1b-1*) had significantly lower stomatal conductance levels than controls during stomatal opening in response to increasing light intensity. However *apk1b-1* conductance levels were not significantly lower during dark-induced stomatal closure (**Figure 2**). This finding was consistent with the results of stomatal aperture measurement experiments showing that stomata of *apk1b-1* and *apk1b-2* were both less open than control stomata in the light, and also in the presence of other stimuli such as ABA and CO_2_ in the light (**Figure 4**). These stomatal aperture measurements suggested that guard cell ABA and CO_2_ signaling pathways are not affected in *apk1b-1* and *apk1b-2* as both mutant genotypes adjust their stomata to a similar extent in response to these stimuli. Both mutants also closed their stomata to the same extent as controls in the dark. In addition, lack of APK1b had no effect on stomatal size, stomatal density or stomatal index (**Figure 3**) indicating that APK1b is not required for stomatal development.

The smaller stomatal apertures that we observed in *apk1b* mutants in the light suggest that these plants would be expected to lose water less readily, and indeed their leaf temperatures were increased following drought stress indicating reduced transpiration. In addition, the relative water content of both *abk1b* genotypes was found to be significantly higher than control plants when plants were either well-watered or following one week of drought (**Figure 5**). Thus, *apk1b* leaves have an enhanced ability to retain water over controls, and therefore might be expected to have increased water use efficiency and tolerance to drought conditions.

Taken together the results discussed above indicate that *APK1b* protein kinase is involved in mediating stomatal opening in the light. Stomata are responsive to red and blue light wavelengths. They open gradually in response to red light, but rapidly and strongly in response to a blue light signal [17]. Protein phosphorylation is known to be important in the blue light-induced stomatal opening pathway. Blue light perception by phototropins causes the multiple phosphorylation of phototropins themselves, the phosphorylation of BLUS1 kinase [6] and ultimately the phosphorylation and activation of the guard cell plasma membrane H^+^-ATPase. The pumping of protons out of guard cells causes membrane hyperpolarization, activating plasma membrane channels that take up potassium ions (K^+^_in_ channels), thus bringing about an increase in turgor pressure that results in stomatal opening. The identity of the kinase that phosphorylates and activates the H^+^-ATPase is not known although its activity has been detected in plasma membrane fractions [18]. We investigated the possibility that the *APK1b* protein kinase may affect the phosphorylation cascade that regulates the activity of the H^+^-ATPase triggering blue light-induced stomatal opening but we could not identify a specific impairment in *apk1b* blue light-induced opening. It therefore appears likely that *APK1b* acts elsewhere in the light-induced stomatal opening pathway, or perhaps has a more general effect on the mechanism of stomatal opening.

## Materials and Methods

### Plant Growth

*Arabidopsis thaliana* mutants were identified from the SALK collection [19] and seed obtained from the Nottingham Arabidopsis Stock Centre (Salk_001115 and Salk_104202 respectively). Columbia (Col-0) background ecotype and mutant seeds were stratified on M3 compost for 72 hours in the dark at 4°C before transfer to a growth room with 10 hours photoperiod, 140 µmol m^−2 ^s^−1^ light intensity, 20°C/16°C day/night temperature, relative humidity 60%. Plants were grown in 8 cm diameter pots and sub-irrigated every three days, until rosettes were mature but flowering had not commenced. Fully expanded leaves were used for IRGA, relative water content and stomatal measurement analyses.

### P*_APK1b_*: GUS Analysis

A region approximately 1200 bp upstream of the ATG promoter translational start codon in *APK1b* was amplified from genomic DNA using forward 5′-CACCTGCTTTTACTTTTCAGGTGCCTA-3′ and reverse primers 5′-GGAGTGAATTAAACCAAACACCA-3′ and KOD DNA polymerase, inserted into the pENTR-D-TOPO entry vector (Invitrogen), transformed into *E. coli* competent cells and recombined with the pHGWFS7 destination vector [20], before transfer into *Agrobacterium tumefaciens* cells and transformation into Col-0 Arabidopsis by floral dip [21]. Seeds were selected on 20 µgml^−1^ hygromycin and insertion confirmed by PCR using forward *APK1b* specific primer and GUS gene reverse primer (5′-TGCTCAGGTAGTGGTTGTCG-3′). Histochemical staining for GUS activity was carried out on leaves of T2 seedlings in 50 mM potassium phosphate, 1 mM potassium ferrocyanide, 1 mM potassium ferricyanide, 0.2% Triton X-100, 2 mM 5-bromo-4-chloro-3-indolyl-β-d-glucuronic acid, and 10 mM EDTA after vacuum infiltration at 37°C. Leaves were decolorized overnight with 70% (v/v) ethanol, and washed in 10% glycerol. Images were captured with an Olympus BX51 microscope connected to a DP51 digital camera. Expression pattern shown was typical of several independently transformed lines.

### Characterisation of Homozygous Knockout Plants

Homozygous plants were identified using primers APK1b-1F: 5′-TCTGAGTCGTGTAAACGAGCC-3′ APK1b-1R: 5′-CCTTTATCTTGGACTCTCCGG-3′, APK1b-2F: 5′-TCTGAGTCGTGTAAACGAGCC-3′and APK1b-2R: 5′-CCTTTATCTTGGACTCTCCGG-3′. Primers APK1b-1F and APK1b-1R were used to amplify the cDNA of *apk1b-1* and *apk1b-2*. RNA was extracted from seedlings using a Qiagen RNAeasy kit, and RNA reverse transcribed into cDNA using oligo dT primer and SuperscriptII (Invitrogen). *UBQ10* amplification was used as a loading control. No DNA amplification was detected with APK1b specific primers after 35 thermocycles.

### Gas Exchange Analysis

Infra-red gas analysis was applied to investigate carbon assimilation, intercellular CO_2_ concentration, and stomatal conductance using a Li-COR 6400 system (Lincoln, NE, US). Measurements were taken from four leaves (attached to the plant) from four separate plants of each genotype. The leaf chamber (2 cm^−2^) was maintained at 20°C. Flow rate was 500 µmol m^−2 ^s^−1^ and humidity (58%–63%).

### Stomatal Density and Index

Dental resin (Coltene Whaledent, Switzerland) was applied to leaf adaxial surfaces. Nail varnish peels were taken from set resin and mounted on microscope slides. Cell counts were taken from the widest area of 3 leaves each from 3 plants of each genotype. Three different areas from each impression were examined under the light microscope with an eyepiece grid. The number of stomata and epidermal cells per square millimeter were obtained to calculate stomatal density, and stomatal index using the equation stomatal index = stomatal number/(stomatal number + epidermal cell number) ×100.

### Stomatal Aperture Analysis

The stomatal of abaxial leaf epidermal strips were analysed using a method described [22]. Strips of epidermis were taken from leaves of five to six week-old plants (3–5 leaves of each genotype) using tweezers and then floated on resting buffer (10 mM MES, pH 6.2). Strips were transferred to opening buffer (10 mM MES, 50 mM KCL, pH 6.2) in the light (300 µmol m^−2 ^s^−1^), aerated with CO_2_-free air and maintained at 20°C. To investigate the effect of ABA on stomatal aperture, epidermal peels were incubated on resting buffer for 10 minutes and then transferred to opening buffer supplemented with ABA concentrations as indicated, and exposed to light and CO_2_ free air for 2 hours. To investigate the effect of CO_2_, epidermal peels were exposed to CO_2_-free or ambient air in light for two hours. Digital images were recorded for stomatal aperture width (and length) measurements by light microscopy (Olympus BX51; Tokyo) using a fitted camera and graticule. For each time point or treatment and genotype, 40 pore apertures were measured. To avoid experimenter bias, measurements were performed without the researcher being aware of the sample identity. Each experiment was then repeated on three separate days using fresh plants (n = 120).

### Relative Water Content

8–10 mature leaves were excised from well-watered or droughted plants (water withheld for 7 days) and their fresh weight measured immediately. Leaves were floated on water at 4°C overnight and weighed to record the hydrated weight. They were oven-dried at 70°C overnight and weighed to obtain their dry weight; the RWC was calculated using the following formula [23]: RWC = (fresh weight−dry weight)/(hydrated weight−dry weight).

### Thermal Imaging

8 week old well-watered plants were subjected to drought for 12 days; plants were then watered normally for a further 6 days. Plants were imaged every 2 or 3 days between 1 and 2 pm with a FLIR SC660 thermal imaging camera. Images were analysed using ThermaCAM Researcher Professional 2.9. For each image, the mean temperature from spots in the centre of 3 representative leaves was calculated.

### Statistical Analyses

Values were statistically tested using unpaired t-tests (**Figures 2** **,** **3** **,** **4** **,** **5A, S** **4**) or ANOVA (**Figure 5B**).

## Supporting Information

Figure S1 ***APK1B*** **shows similar expression patterns to the know guard cell gene** ***OST1*** **, and is enriched in guard cell preparations.** Raw expression values were extracted from the manually dissected guard cells (A;S1), or protoplasts (B,C;S2,S3). *OST1* is expressed in guard cells in leaves (S4) whereas *STOMAGEN* (*STOM*) is mesophyll specific (S5).
(TIF)

Figure S2 ***pAPK1B: GUSGFP*** **is predominantly expressed in mature guard cells.** Fluorescence of 14 day old seedlings. A Abaxial fluorescence, showing mature guard cells with fainter fluorescence in developing guard cells. Bar = 50 m B Adaxial fluorescence, showing mature guard cells in developing leaf. Bar = 1 mm C Adaxial fluorescence, showing mature guard cells in developing leaf Bar = 100 m. D. GUS expression in cauline leaves, flowers and stem. Bar = 2 cm.
(TIF)

Figure S3 ***apk1b-1*** **and** ***apk1b-2*** **are null mutants.** cDNA was synthesised from RNA extracted from seedlings of the T-DNA disruption mutant *apk1b-1* and *apkb1-2* and Col-0 control and amplified with gene specific primers, for 35 cycles before electrophoresis. No wild-type *APK1b* transcript was amplified from either mutant.
(TIF)

Figure S4 **Photosynthesis and intracellular CO_2_ in** ***apk1b-1*** **mutants.** IRGA photosynthetic parameters measured by IRGA of leaves. A, C & E: Assimilation rate; B, D & F: intercellular CO_2_ levels. A & B: Leaves of plants exposed to saturating light for 30 min, then exposed to darkness, arrow indicates when light was switched off. C & D: Leaves exposed to increasing light intensities ranging from 0 to 2000 µmol photon m^−2 ^s^−1^. E & F: Leaves of plants exposed to red light for 60 min, then exposed to blue and red light, arrow indicates when blue light is switched on. Error bars represent the standard errors. Values were statistically tested using unpaired t-tests and significant differences are indicated (** = p<0.01).
(TIF)

File S1 **Supporting Information References.**
(DOCX)
