# Supplementary figures and images for: Light-Induced Stomatal Opening Is Affected by the Guard Cell Protein Kinase APK1b
Source: PLoS One. 2014 May 14;9(5):e97161. doi: 10.1371/journal.pone.0097161 (PMC4020820; doi:10.1371/journal.pone.0097161)

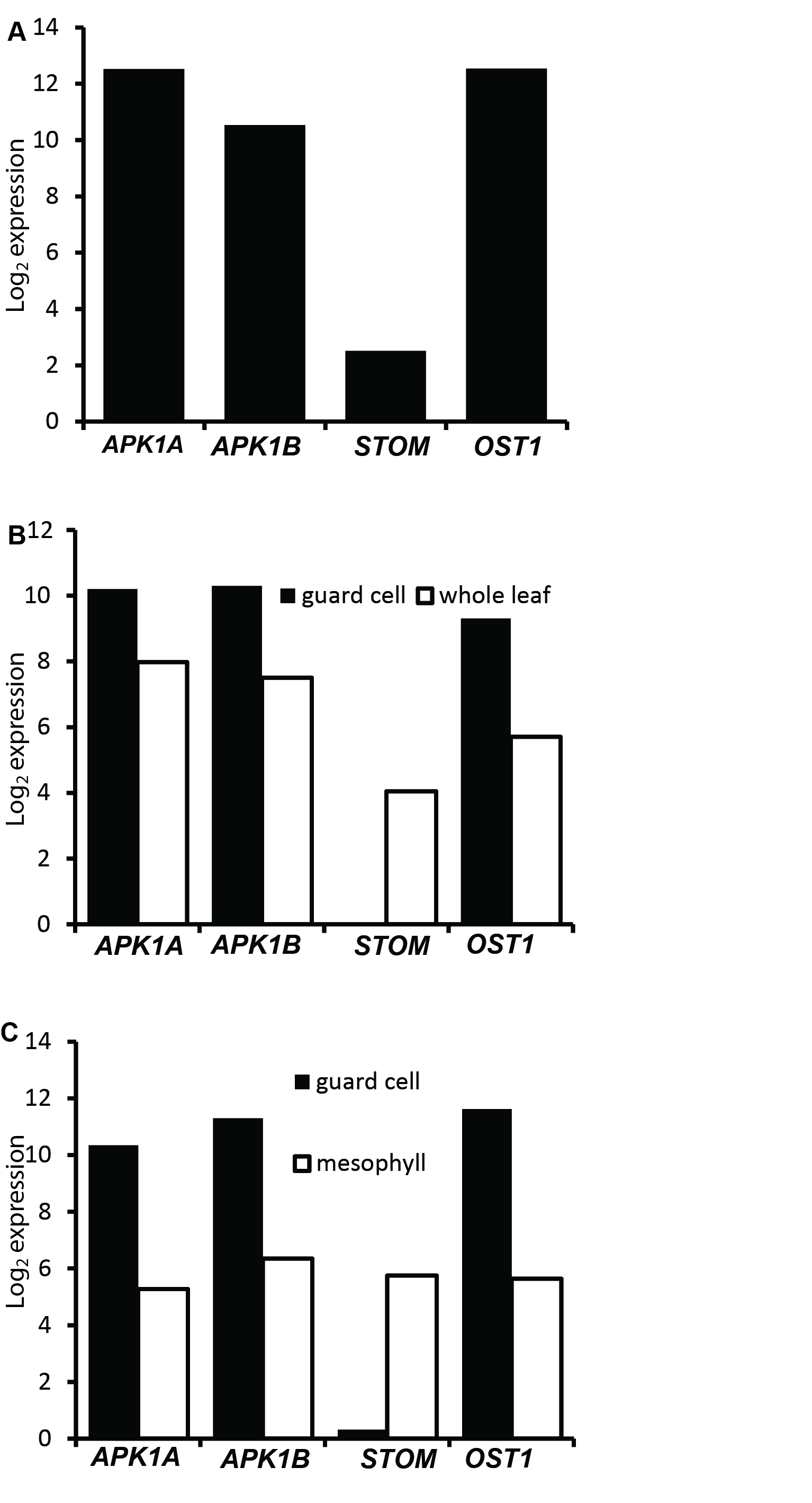

Supplement: Figure S1 — APK1B shows similar expression patterns to the know guard cell gene OST1 , and is enriched in guard cell preparations. Raw expression values were extracted from the manually dissected guard cells (A;S1), or protoplasts (B,C;S2,S3). OST1 is expressed in guard cells in leaves (S4) whereas STOMAGEN (STOM) is mesophyll specific (S5). (TIF) [file pone.0097161.s001.tif]

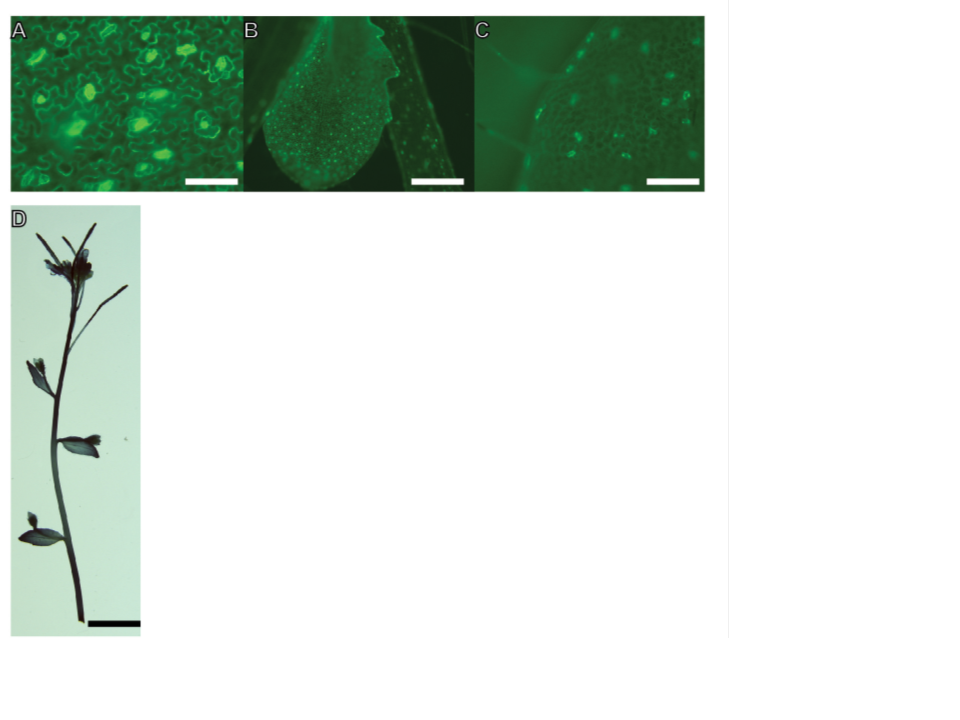

Supplement: Figure S2 — pAPK1B: GUSGFP is predominantly expressed in mature guard cells. Fluorescence of 14 day old seedlings. A Abaxial fluorescence, showing mature guard cells with fainter fluorescence in developing guard cells. Bar = 50 m B Adaxial fluorescence, showing mature guard cells in developing leaf. Bar = 1 mm C Adaxial fluorescence, showing mature guard cells in developing leaf Bar = 100 m. D. GUS expression in cauline leaves, flowers and stem. Bar = 2 cm. (TIF) [file pone.0097161.s002.tif]

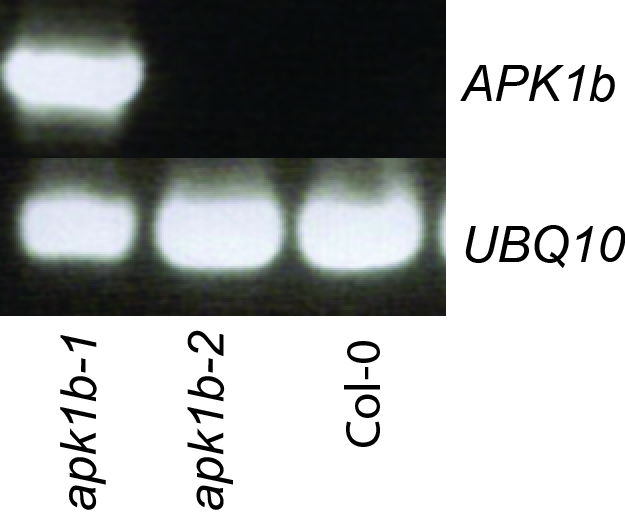

Supplement: Figure S3 — apk1b-1 and apk1b-2 are null mutants. cDNA was synthesised from RNA extracted from seedlings of the T-DNA disruption mutant apk1b-1 and apkb1-2 and Col-0 control and amplified with gene specific primers, for 35 cycles before electrophoresis. No wild-type APK1b transcript was amplified from either mutant. (TIF) [file pone.0097161.s003.tif]

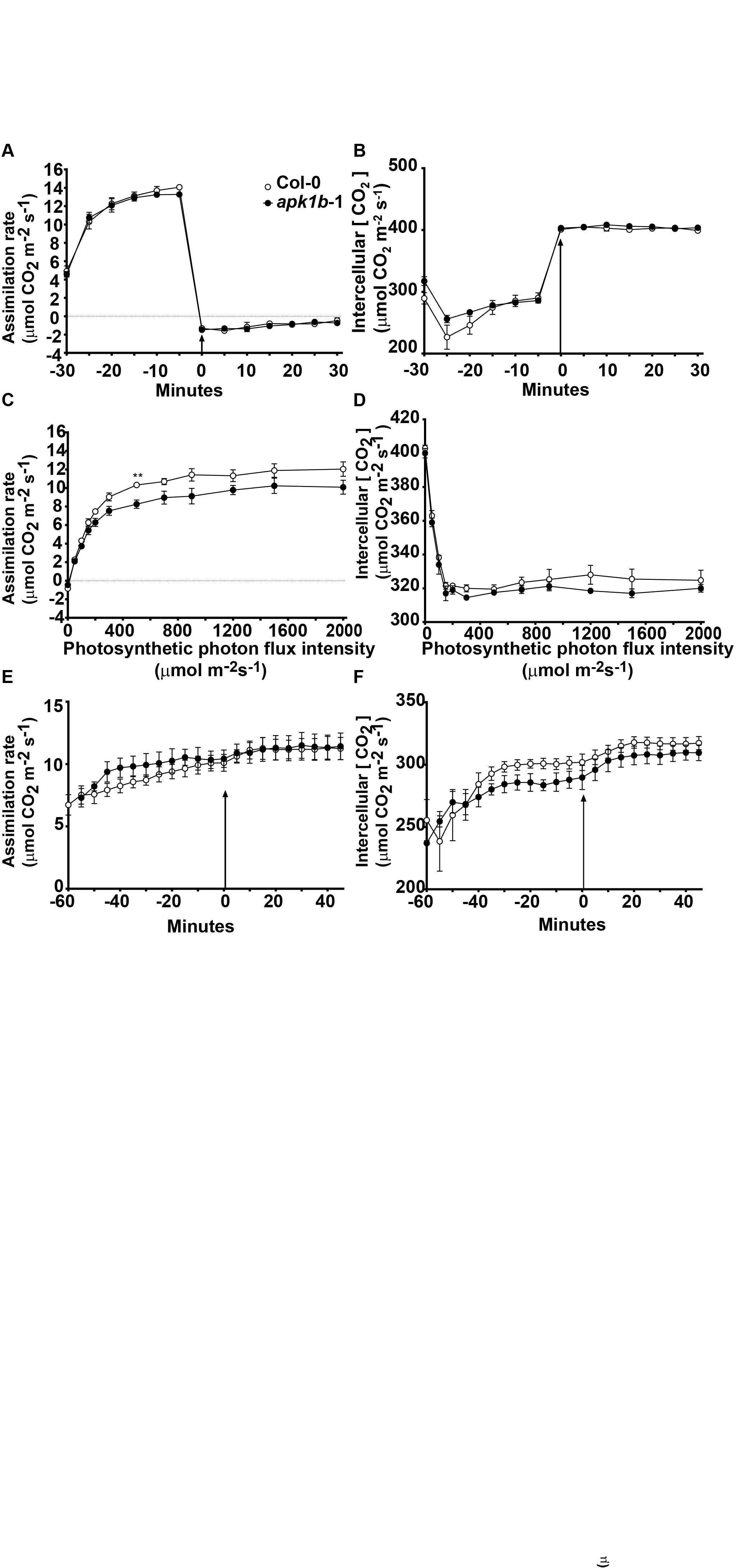

Supplement: Figure S4 — Photosynthesis and intracellular CO2 in apk1b-1 mutants. IRGA photosynthetic parameters measured by IRGA of leaves. A, C & E: Assimilation rate; B, D & F: intercellular CO2 levels. A & B: Leaves of plants exposed to saturating light for 30 min, then exposed to darkness, arrow indicates when light was switched off. C & D: Leaves exposed to increasing light intensities ranging from 0 to 2000 µmol photon m−2 s−1. E & F: Leaves of plants exposed to red light for 60 min, then exposed to blue and red light, arrow indicates when blue light is switched on. Error bars represent the standard errors. Values were statistically tested using unpaired t-tests and significant differences are indicated (** = p<0.01). (TIF) [file pone.0097161.s004.tif]
