# Supplementary material for: Light-Induced Stomatal Opening Is Affected by the Guard Cell Protein Kinase APK1b
Source: PLoS One. 2014 May 14;9(5):e97161. doi: 10.1371/journal.pone.0097161 (PMC4020820; doi:10.1371/journal.pone.0097161)
Supplement: File S1 — Supporting Information References. (DOCX) [file pone.0097161.s005.docx]

**Supporting Information References**

**S1** Bates GW, Rosenthal DM, Sun J, Chattopadhyay M, Peffer E, Yang J, Ort DR, Jones AM (2012) A comparative study of the Arabidopsis thaliana guard-cell transcriptome and its modulation by sucrose. PLoS One 7: e49641. doi: 10.1371/journal.pone.0049641.

**S2** Pandey S, Wang RS, Wilson L, Li S, Zhao Z, Gookin TE, Assmann SM, Albert R (2010) Boolean modeling of transcriptome data reveals novel modes of heterotrimeric G-protein action. Mol Syst Biol 6: 372.

**S3** Yang Y, Costa A, Leonhardt N, Siegel RS, Schroeder JI (2008) Isolation of a strong Arabidopsis guard cell promoter and its potential as a research tool. Plant Methods 4: 6.

**S4** Mustilli, AC, Merlot S, Vavasseur A, Fenzi F, Giraudat J (2002) Arabidopsis OST1 protein kinase mediates the regulation of stomatal aperture by abscisic acid and acts upstream of reactive oxygen species production. Plant Cell 14:3089-3099.

**S5** Sugano SS, Shimada T, Imai Y, Okawa K, Tamai A, Mori M, Hara-Nishimura I (2010) Stomagen positively regulates stomatal density in Arabidopsis. Nature 14: 463:241-244.
